# Correlation Analysis between Mechanical Power and Lung Ultrasound Score and Their Evaluation of Severity and Prognosis in ARDS Patients

**DOI:** 10.1155/2021/4156162

**Published:** 2021-09-01

**Authors:** Yongpeng Xie, Suxia Liu, Zhifang Mou, Yanli Wang, Xiaomin Li

**Affiliations:** ^1^Department of Critical Care Medicine, Lianyungang Clinical College of Nanjing Medical University, the First People's Hospital of Lianyungang, Lianyungang, China; ^2^Department of Emergency Medicine, Lianyungang Clinical College of Nanjing Medical University, the First People's Hospital of Lianyungang, Lianyungang, China

## Abstract

**Methods:**

A total of 121 patients with moderate to severe ARDS admitted to the intensive care unit (ICU) from June 2017 to April 2020 and treated with invasive mechanical ventilation were sequentially included in this study. Their general information was collected, and MP was recorded at 0 h, 24 h, 48 h, and 72 h after admission to the ICU. Professionally trained researchers performed the LUS assessments. Patients were divided into the death and survival groups according to their 28-day prognosis. The trend of MP and LUS at the four time points was analyzed. A receiver operating characteristic curve (ROC) was used to analyze the predictive value of MP and LUS scores at 0 h and 72 h for the prognosis (28-day mortality rate) of patients with moderate to severe ARDS.

**Results:**

121 patients were included in the analysis, of which 73 were male and 48 were female. When patients entered the ICU, their oxygenation index (*t*: 30885, *P* < 0.01), APACHE II score (*t*: 2.105, *P* < 0.05), and SOFA score (*t*: 4.134, *P* < 0.001) were higher in the death group than the survival group. The death group had significantly higher MP and LUS at each time point (0 h, 24 h, 48 h, and 72 h) compared to the survival group (all *P* < 0.05). There was a significant upward trend over time in the MP and LUS of the death group, contrasting to a significant downward trend in the survival group (all *P* < 0.05). The Pearson correlation analysis showed that MP and LUS were significantly positively correlated at each time point (*r* values: 0 h: 0.3027; 24 h: 0.3705; 48 h: 0.3902; 72 h: 0.5916; all *P* < 0.01). The ROC curves showed that MP and LUS at 72 h were of significant value in predicting the prognosis of ARDS patients, with areas under the curve of 0.866 ± 0.032 and 0.839 ± 0.037, respectively.

**Conclusion:**

There was a significant correlation between the MP and LUS of ARDS patients at four time points from 0 to 72 h, which has a clinical value in evaluating severity and prognosis.

## 1. Introduction

Acute respiratory distress syndrome (ARDS) is one of the most common critical illnesses in intensive care medicine. An international epidemiological study (LUNG SAFE) reported that ARDS incidence in the ICU is about 10% [1]. It is characterized by high morbidity and mortality rates, and as such, it is a major medical problem [2]. However, the mortality of ARDS has witnessed a declining trend in the past few decades owing to strenuous efforts made by the critical care community [3].

Gattinoni et al. [4] verified the accuracy of the Berlin standard of ARDS and found that parameters of respiratory mechanics during mechanical ventilation provide a meaningful tool for evaluating the severity and prognosis of ARDS. Specifically, the concept of mechanical power (MP) has received attention from clinicians. Through clinical and animal studies, the assessment of mechanical power has been confirmed as an effective means of evaluating the prognosis and severity of ARDS, especially the optimal MP values estimated by a dynamic treatment regime model are another better way than the gloss MP to show the association between MP and mortality outcome [5–7]. Improvements in pulmonary ventilation directly affect the prognosis of ARDS patients. Advances in lung ultrasound technology have made it possible to evaluate ventilation at the bedside [8], and further analysis by semiquantitative image scoring is more convenient than other methods such as computed tomography examinations. Wang et al. [9] used lung ultrasound scores (LUS) to assess pulmonary ventilation status, confirming that lung ultrasound can be used to evaluate ARDS prognosis.

The accurate assessment of ARDS severity at an early stage and identifying appropriate measures to reduce the mortality rate and improve the prognosis of ARDS patients has long been the focus of critical care medicine [10]. This study examined the correlation between the early mechanical powers of mechanical ventilation with lung ultrasound scores in patients with ARDS. The prognostic value of mechanical power and lung ultrasound scores were also explored.

## 2. Research Participants

### 2.1. Recruitment

Patients with moderate to severe ARDS, who required mechanical ventilation for longer than 48 hours, were sequentially selected from patients admitted to the First People's Hospital of Lianyungang from June 2017 to June 2020. Participants were included in the study if they were ≥18 years of age and their condition complied with the 2012 Berlin definition diagnostic criteria for moderate to severe ARDS, including P/F ≤ 200 mmHg [11], treatment with invasive mechanical ventilation, and an estimated treatment period of more than 48 hours. Patients were excluded if they were less than 18 years of age, and if they were pregnant; had chest trauma or bandaging of chest wall, thoracic deformity, pneumothorax, massive pleural effusion, a history of pulmonary alveolar disease, severe hemodynamic abnormalities, severe heart failure, and acute coronary syndrome; and patients undergoing extracorporeal membrane oxygenation (ECMO) treatment.

### 2.2. Ethics Statement

The study was approved by the ethics committee of the Lianyungang Clinical College of Nanjing Medical University (approval number: LCYJ20170312001). Before participating in the study, written informed consent was obtained from the patient's legal representatives. Patient information was anonymized and deidentified prior to analysis. This project was registered in the China Clinical Trial Registration Center (registration number: ChiCTR1900028238).

## 3. Research Methods

### 3.1. Mechanical Ventilation

The basic information of all patients was recorded, as well as the sequential organ failure assessment (SOFA) score and the 24-hour acute physiology and chronic health evaluation (APACHE) II score. And they were ventilated according to the original ARDSnet protocol [12]. Briefly, patients were ventilated in a volume-assist control mode with constant square flow and a tidal volume of 6 mL/kg of ideal predicted body weight. The goal of oxygenation was to target a peripheral blood oxygen saturation (SpO_2_) between 88% and 95% measured by pulse oximetry, or a partial pressure of oxygen (PaO_2_) of 55–80 mmHg measured by arterial blood gas analysis. To achieve stable oxygenation, the fraction of inspired oxygen (FiO_2_) and positive end-expiratory pressure (PEEP) were adjusted as described in previous studies (ARMA and ACURASYS studies) [13]. The respiratory rate was adjusted to ensure the arterial pH was between 7.20 and 7.45.

### 3.2. Mechanical Power in ARDS Patients

All patients in this study underwent mechanical ventilation with tracheal intubation and were given analgesia and sedation. Parameters of respiratory mechanics were recorded after 0 h, 24 h, 48 h, and 72 h of mechanical ventilation and included driving pressure (DP), respiration rate (RR), peak airway pressure (Ppeak), and tidal volume (VT). Mechanical power (J/min) was calculated according to the classical simplified formula: MP = 0.098 × RR × VT × (Ppeak − 1/2DP) [6]; the average of three measurements was taken as MP at each time point.

### 3.3. Lung Ultrasound Score at the Bedside

To ensure a complete assessment of lung function, all patients were subjected to a lung ultrasound examination scored by two specially trained ultrasound researchers at 0 h, 24 h, 48 h, and 72 h of mechanical ventilation. A total of 12 lung areas, bilaterally encompassing the front, sides, and back (upper and lower parts) of the chest wall, were examined, and video data were saved. The following criteria were used to score ultrasound images [9]: (1) normal ventilation zone (N): signs of lung sliding with A-line or less than two separate B-lines; (2) moderately reduced lung ventilation (line B1): multiple, typical B-lines; (3) severely reduced pulmonary ventilation (line B2): B-lines with multiple fusions; and (4) lung consolidation (C): tissue image shows typical signs of bronchial inflation. Each area was scored from zero (N) to three (C), according to the most severe rating observed. The LUS was taken as the sum of the scores from each area, with a total score ranging from 0 to 36 points.

### 3.4. Grouping and Statistical Analysis

Data processing and mapping were performed using SPSS 22.0 statistical software and GraphPad Prism 6.0. The patients were divided into the death and survival groups according to their 28-day prognosis. MPLU 8.13 was used to conduct latent growth modeling (LGM) analysis on time-varying trends of MP and LUS [14]. The data are represented by the mean and standard deviation (*x* ± *s*), and the difference between the two groups was compared using two independent samples *t*-tests. The *χ*^2^ test was used to compare categorical data. The area under the receiver operating characteristic curve (AUC) was used to analyze the predictive value of MP and LUS after 0 h and 72 h of treatment for estimating the 28-day survival status of patients with moderate to severe ARDS. A *P* value of < 0.05 was considered statistically significant.

## 4. Results

A total of 153 patients with moderate to severe ARDS were screened. Twenty-four cases met the exclusion criteria and were removed, leaving 129 cases that were included in the study. During the experiment, eight patients were excluded due to acute left heart failure or other severe hemodynamic disorders. Finally, data from 121 patients were included in the analysis, of which 73 were male and 48 were female.

### 4.1. General Clinical Data

The survival (75 cases) and death groups (46 cases) were similar in sex, age, body mass, body mass index, basic medical history, and smoking history (all *P* > 0.05). However, the APACHE II, SOFA, PaO_2_/FiO_2_, and blood lactic acid were significantly different between the groups (all *P* < 0.05; Table 1).

### 4.2. Mechanical Power and Lung Ultrasound Scores

**Figure 1:**
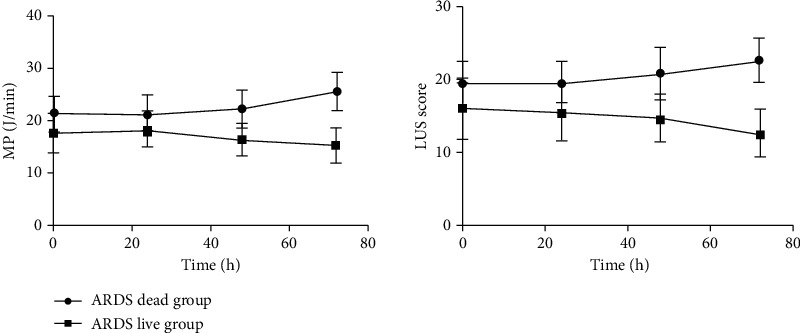
Trends in MP and LUS between the two groups over the four time points.

The analysis results of the LGM show that the intercept represents the starting level of the MP/LUS trend of each group, and the slope represents the change speed of the MP/LUS development trend. Over time, the MP and LUS of the survival group showed a significant downward trend, while an upward trend was observed in the death group (all *P* < 0.05; Table 2 and Figure 1).

**Figure 2:**
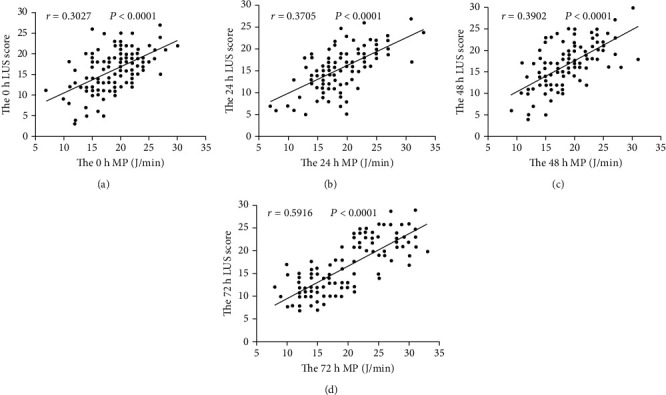
Correlation analysis of MP and LUS at the four time points.

In all patients, MP and LUS were significantly positively correlated at 0 h (*r* = 0.3027), 24 h (*r* = 0.3705), 48 h (*r* = 0.3902), and 72 h (*r* = 0.5916) (all *P* < 0.0001; Figure 2).

### 4.3. Evaluating Prognosis with MP and LUS

**Figure 3:**
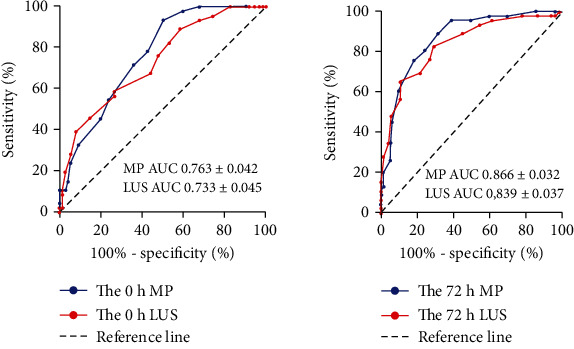
ROC curve of MP and LUS for prognostic evaluation of patients at 0 hours and 72 hours.

The ROC curve analysis showed that the area under the curve of MP and LUS on admission to the intensive care unit were 0.763 ± 0.042 and 0.733 ± 0.045, respectively. The area under the curve of MP and LUS measured at 72 h after treatment were 0.866 ± 0.032 and 0.839 ± 0.037, respectively, and the predictive value was significant (Figure 3).

### 4.4. 28-Day Survival Status

**Figure 4:**
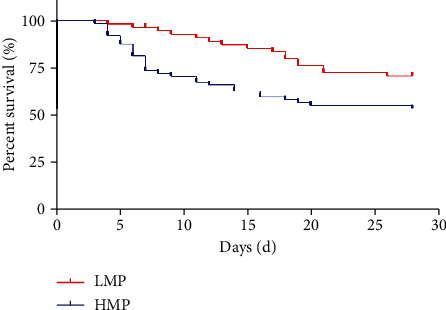
Mechanical power and 28-day survival curve.

The patients were divided into high mechanical power (HMP; 65 cases) and low mechanical power (LMP; 56 cases) groups according to whether their MP at 72 h exceeded 17 J/min. As 46 cases died in 28 days, analyzing the Kaplan-Meier survival curve revealed that the overall survival rate was 61.98%. The survival rate was significantly lower in the HMP group (53.85%) than in the LMP group (71.43%; *P* < 0.05; Figure 4).

## 5. Discussion

With advances in the understanding of ARDS, new treatment methods and concepts are constantly being introduced [15]. However, mechanical ventilation remains the most important treatment strategy for ARDS patients. Whether it is achieved through spontaneous breathing or mechanical ventilation, lung ventilation is the mechanical process by which the power of breathing overcomes airway resistance and drives gas exchange between the lungs and environment [16, 17]. Therefore, an in-depth understanding of the respiratory mechanics of ARDS patients during mechanical ventilation has important clinical significance for exploring its pathophysiological mechanisms, formulating individualized lung-protective ventilation plans, assessing the severity of the patient's condition, and predicting the patient's prognosis. The concept of mechanical power [18], as a parameter of lung energy load, provides a more comprehensive understanding of the biomechanical interactions between the ventilator and the lung tissue in the ARDS treatment process [19, 20].

The examination of lung ultrasound images of critically ill patients has been widely used in many fields of intensive care medicine because it is noninvasive, repeatable, radiation-free, and easily performed by clinicians [21]. For patients with moderate to severe ARDS, an early, accurate, and rapid assessment to determine lung ventilation status and lung water content have very important clinical significance for reducing mortality and complications [22]. In addition, the quantitative scoring of lung ultrasound images provides an effective means for evaluating the severity of lung disease in patients with ARDS.

This study observed dynamic changes in MP and LUS among patients with ARDS after 0, 24, 48, and 72 hours of mechanical ventilation. Over this period, MP and LUS showed a significant downward trend in the survival group. On the contrary, the death group showed an upward trend. At each time point, MP and LUS were significantly higher in the death group. These findings indicate that as a patient's condition improves (survival group), their pulmonary edema is gradually relieved, their pulmonary ventilation status increases, and their respiratory mechanics manifest a progressive decline in MP. Alternatively, the disease became progressively worse in the death group. Pulmonary edema became worse over time, the area of lung ventilation gradually decreased or was lost, and MP progressively increased. Higher LUS in the death group represents more severe alveolar collapse, pulmonary edema, and lung consolidation [23]. In the process of mechanical ventilation, in order to maintain the collapsed lungs in an open state, the ventilator requires more mechanical power [19, 24]. In terms of the conventional mechanical parameters, greater lung driving pressure and positive end-expiratory pressure are needed to maintain alveolar opening [25, 26]. In other words, a higher LUS of the lungs in ARDS indicates greater mechanical power requirements during mechanical ventilation. Therefore, the two parameters are closely related to the severity of the patient's lung lesions. There was a clear positive correlation between the MP and LUS at each time point, and these indicators were also correlated with the pathophysiological outcome of the disease. The stronger the correlation, which also reflects the severity of ARDS, confirms that MP and LUS have the same pathophysiological basis. Therefore, both theoretically and clinically, there is a close relationship between the two parameters. However, the association between MP/LUS and mortality cannot be understood as causal association, that is, we may not improve mortality outcome by reducing MP/LUS.

In this study, through receiver operating characteristic curve analysis, the area under the curves of MP and LUS at 72 h was 0.866 ± 0.032 and 0.839 ± 0.037, and they had a significant predictive value. At the same time, the survival curve of patients with ARDS indicated that there was a statistically significant difference in 28-day mortality between the HMP and LMP groups. These findings confirm that monitoring the MP and LUS of ARDS patients is of great significance in evaluating the severity of ARDS lung injury and predicting patient prognosis [27]. These findings provide an effective basis for doctors in critical care medicine to make accurate clinical decisions regarding ARDS.

## 6. Conclusions

There is a significant correlation between MP and LUS in the early stages of ARDS. Comprehensively controlling the MP within the target range by adjusting VT, limiting DP, or reducing RR provides an objective clinical basis for guiding standardized and precise interventions for ARDS as early as possible.

## Figures and Tables

**Table 1:** Baseline characteristics of the two groups.

Index/group	Survival group (*n* = 75)	Death group (*n* = 46)	$\frac{t}{x^{2}}$	*P*
Gender (M/F)	44/31	29/17	0.478	0.633
Weight (kg)	62.51 ± 9.34	63.48 ± 10.43	0.530	0.597
BMI (kg/m^2^)	24.35 ± 3.28	24.47 ± 4.23	0.175	0.861
SOFA	8.48 ± 2.90	10.61 ± 2.83	4.134	0.000
APACHEII	21.53 ± 6.59	23.91 ± 5.91	2.105	0.037
PaO2/FiO2 (mmHg)	139.56 ± 24.38	119.58 ± 31.89	3.885	0.002
LAC (mmol/L)	7.66.±2.55	7.59 ± 2.51	1.749	0.084
Smoking (Y/N)	23/51	17/29	0.664	0.507
Basic medical history (Y/N)	34/43	20/26	0.073	0.942

Note: the basic medical history includes hypertension, diabetes, coronary heart disease, and history of trauma surgery.

**Table 2:** Analysis of LGM of MP and LUS between the two groups at four time points.

	Group	CFI	RASEA	SRMR	*I*	*S*	*x* ^2^	*P*
MP	Survival group (*n* = 75)	0.790	0.180	0.151	18.223	-0.866	17.133	0.004
	Death group (*n* = 46)	0.573	0.352	0.564	20.696	1.041	72.522	0.000
LUS	Survival group (*n* = 75)	0.866	0.253	0.260	15.870	-1.109	29.051	0.000
	Death group (*n* = 46)	0.920	0.170	0.132	18.739	1.137	11.633	0.040

## Data Availability

Data are available on request.
